# Metformin Inhibits Expression and Secretion of PEDF in Adipocyte and Hepatocyte via Promoting AMPK Phosphorylation

**DOI:** 10.1155/2013/429207

**Published:** 2013-10-31

**Authors:** Shumin Yang, Qiong Lv, Ting Luo, Lulu Liu, Rufei Gao, Shumei Chen, Peng Ye, Qingfeng Cheng, Qifu Li

**Affiliations:** Department of Endocrinology, The First Affiliated Hospital of Chongqing Medical University, No. 1 Youyi Street, Chongqing 400016, China

## Abstract

*Objective*. Pigment epithelium-derived factor (PEDF) plays an important role in obesity-induced insulin resistance (IR). The study aims to investigate the effect of metformin, a widely used agent to improve IR, on PEDF production both in vivo and in vitro. *Methods*. SD rats were divided into normal control group, high fat group (HF group), and metformin group (MET group). Hyperinsulinemic euglycemic clamp was performed to evaluate insulin sensitivity. IR models of 3T3-L1 and HepG2 cells were established and then treated with metformin and inhibitor of AMP activated protein kinase (AMPK). *Results*. In vivo, the HF group showed increased serum PEDF which is negatively correlated with insulin sensitivity, while the MET group revealed decreased serum PEDF and downregulated PEDF expression in fat and liver, concomitant with significantly improved IR. In vitro, the IR cells showed enhanced PEDF secretion and expression, whereas metformin lowered PEDF secretion and expression, accompanied with increased glucose uptake. Metformin stimulated AMPK phosphorylation in fat and liver of the obese rats, while in vitro, when combined with AMPK inhibitor, the effect of metformin on PEDF was abrogated. *Conclusions*. Metformin inhibits the expression and secretion of PEDF in fat and liver via promoting AMPK phosphorylation, which is closely associated with IR improvement.

## 1. Introduction

Metformin is widely used to improve insulin resistance (IR), but the mechanism is not fully understood. Although liver is the primary target, metformin acts on a variety of tissues, including adipose tissue, skeletal muscles, endothelium, and ovary [1, 2]. Previous studies have demonstrated metformin exerts the metabolic actions, especially the glucoregulatory action, mainly through AMP activated protein kinase (AMPK) [3, 4]. In recent years, metformin has been reported to have an impact on the production of adipokines (e.g., adiponectin, leptin), which might be associated with the insulin-sensitizing effect of this agent [5, 6]. 

Pigment epithelium-derived factor (PEDF), an adipokine discovered in recent years, plays an important role in obesity-induced IR [7]. It is one of the most abundant proteins secreted by human adipocytes with the property to induce IR and inflammatory signaling in adipocytes and skeletal cells [8]. Serum PEDF levels elevate in obese mice and reduce upon weight loss; prolonged PEDF administration stimulates adipose tissue lipolysis, results in ectopic lipid deposition, and reduces insulin sensitivity, whereas neutralizing PEDF with PEDF antibody in obese mice enhances insulin sensitivity [7, 9]. Clinical researches have revealed that serum PEDF level is an independent determinant of IR in patients with essential hypertension [10]. In our previous work, increased serum PEDF levels, which are closely associated with IR and chronic inflammation, are observed in patients with polycystic ovary syndrome [11]. Additionally, PEDF is also found to be an important determinant of oxidative stress, inflammation, and angiogenesis [12, 13]. 

It is presently unclear whether metformin could influence PEDF production in adipose tissue and liver. A study showed that the improvement of IR, following diet and/or surgery-induced weight loss, was associated with the magnitude of decrease in serum PEDF levels and PEDF expression of adipose tissue [14]. In this context, the present study was undertaken to get insights into the effect of metformin on the expression and secretion of PEDF in adipocyte and hepatocyte and whether this effect is associated with the IR-improving action of the agent, which may help to clarify pharmacological action of metformin. Our data showed metformin inhibits PEDF production and release, and AMPK plays a key role in this process, which is closely associated with the improvement of IR.

## 2. Methods and Procedures

### 2.1. Animals

Animal care and experimental procedures were performed with the approval from the Animal Care Committees of Chongqing Medical University. After two weeks of adaptive feeding, the seven-week-old SD rats were randomly assigned to receive a normal chow diet (15% fat) (NC group, *n* = 6) or a high fat diet (45% fat) (*n* = 12) for 15 weeks; then the high-fat-diet rats were gavaged saline (HF group, *n* = 6) or metformin (Glucophage, Sanofi, France) 400 mg/kg/d (MET group, *n* = 6) for 4 weeks. At the end of the 19th week, insulin sensitivity was assessed by the hyperinsulinemic-euglycemic clamp, and then the rats were culled with cervical dislocation. When sacrificed, blood and tissue samples were collected for further assessments. 

### 2.2. Cell Culture

Mouse embryo fibroblast 3T3-L1 cells were obtained from American Type Culture Collection (Manassas, VA) and grown in Dulbecco's modified Eagle's medium (DMEM) containing 1% antibiotics (Life Technologies, Maryland, US) as described previously [15]. Human hepatoma cells (HepG2 cells) (obtained from American Type Culture Collection) were grown in 1640 medium supplemented with 10% calf serum and 1% antibiotics. 

The IR cell models were established by the hyperinsulinemic method [16–18]. HepG2 cells and differentiated 3T3-L1 cells seeded on 96-well plates were treated with 0, 0.05, 0.5, 5, and 50 nM insulin for 24 h, and the insulin (100 nM) mediated glucose uptake was examined to validate the establishment of the cell models. 

The IR cell models were treated with or without 0.1 mM metformin (Sigma, US) for 24 h, and the 40 *μ*M compound C (Sigma, US) was added 1 h before metformin addition. Supernatants, RNA, and protein of the cells were collected for PEDF assay.

### 2.3. Measurement of Glucose Uptake

The tracer used to monitor glucose uptake in cells was 6-[N-(7-nitrobenz-2-oxa-1, 3-diazol-4-yl) amino]-6-deoxyglucose (2-NBDG; Sigma, US), with the method described previously, with some modification [19, 20]. After treatment, the cell medium was removed, and the cells were washed twice with Krebs'-Ringer's-HEPES (KRH) buffer (136 mM NaCl, 20 mM HEPES, 4.7 mM KCl, 1.25 mM MgSO_4_, and 1.25 mM CaCl_2_, pH 7.4) and the plate was measured by a fluorescence microplate reader (SpectraMaxM2 multimode plate readers, Molecular Devices, USA) set at an excitation wavelength of 488 nm and an emission wavelength of 520 nm, and the results were expressed as Fa. Treated with 2-NBDG (50 *μ*M) for 15 min and washed twice with KRH buffer, the cells were measured by the fluorescence microplate reader again and the results were expressed as Fb. Later on, treated with 2-NBDG combined with insulin (100 nM) for 15 min and washed twice with KRH buffer, the cells were measured by the fluorescence microplate reader for the third time and the results were expressed as Fc. Finally, the cell viability was measured by 4,5-Dimethylthiazol-2-yl)-2,5-diphenyltetrazolium bromide method and the results were expressed as OD. The increase of fluorescence intensity was calculated as (Fc-Fb)/(Fb-Fa)/OD.

### 2.4. PEDF  and  Metabolite  Analysis

PEDF concentration in the serum and the supernatants of 3T3-L1 and HepG2 cells was analyzed by the ELISA kits (Uscn Life Science Inc, China) according to the manufacturer's instruction. Plasma glucose was assessed by the Hexokinase method (Roche, Switzerland). Serum insulin was determined by the ELISA kits (Uscn Life Science Inc, China). Serum lipids were measured as follows: total cholesterol (TC) using the cholesterol oxidase-HDAOS method (Wako, Osaka, Japan); triglycerides (TG) using the GPO-HDAOS glycerol blanking method (Wako); high-density lipoprotein cholesterol (HDL-c) using the immunoinhibition (direct) method (Wako); and low-density lipoprotein cholesterol (LDL-c) using the selective protection enzymatic (direct) method (Wako). 

### 2.5. Hyperinsulinemic-Euglycemic  Clamps

Clamp was performed as described previously, with some modifications [21, 22]. In brief, after rats were anesthetized with 10% chloral hydrate (0.4 mL/100g body weight), two polyethylene catheters were inserted into the right jugular vein and left carotid artery. Patency was maintained by 0.5% heparin (1 mL/kg body weight) administration, and the vein catheter was connected with two digital syringe pumps (Medtronic, Inc, USA) by a Y connector. Blood samples of 600 *μ*L were obtained for the measurement of serum insulin, lipids, and PEDF before the infusion of insulin. The infusion rate of insulin (Humulin regular insulin, Eli Lilly, USA), mixed with a variable volume of 0.1% BSA, was set at (10 mU/kg/min), and when the plasma glucose was lower than 5.0 mmol/L, 20% glucose infusion began and euglycemia (5.0-6.0 mmol/L) was maintained by a variable infusion rate that was adjusted according to the plasma glucose level at 5 min intervals. During the last 60 min of the clamp when the plasma glucose level became steady, the glucose infusion rate was calculated as the average results of the last thirteen times of plasma glucose levels.

### 2.6. qRT-PCR

Total RNA was extracted using the Trizol reagent (Life Technologies, Maryland, USA) according to the manufacturer's instruction. The RNA purity was assessed by measuring OD at 260 and 280 nm with the standard of A260/A280 ≥ 1.8. RNA integrity was assessed by agarose gel electrophoresis. To eliminate DNA contamination, RNA was treated with 1 unit of DNase I (Life Technologies, Maryland, USA) for 15 min at room temperature. cDNA synthesis was performed with 0.5 mg total RNA treated with DNase I in a 10 *μ*L reaction system using the First Strand synthesis for RT-PCR kit (Takara). cDNA was stored at −80°C until real-time PCR. Real-time reverse transcription polymerase chain reaction (RT-PCR) was performed in the Applied Biosystems 7500 Real-Time PCR System, using SYBR Green dye (QIAGEN, German) according to the manufacturer's protocol. Each quantitative reaction was performed in duplicate. All the primers were designed by Beacon Designer Software and synthesized by Life Technologies Corporation. The specific primers were shown in Table 1. To normalize expression data, actin was used as the internal control gene.

### 2.7. Western Blotting

Proteins were extracted from tissues and cells using cell lysis buffer for western blotting (Beyotime, Jiangsu, China), and protein concentration was determined using the BCA Protein Assay kit (Beyotime). Sample proteins were separated by sodium dodecyl sulfate polyacrylamide gel electrophoresis in a Bio-Rad Mini-Protean apparatus. Then protein extracts were transferred to a PVDF membrane. Membranes were blocked with blocking buffer and incubated with primary antibodies (goat polyclonal anti-PEDF, rabbit polyclonal anti-pAMPK*α*, rabbit polyclonal anti-beta actin, Santa Cruz biotechnology, USA) overnight at −4°C, followed by horseradish peroxidase-labeled secondary antibodies (Santa Cruz biotechnology, USA) for one hour at room temperature. Immunoreactive proteins were visualized using enhanced chemiluminescence (ECL, Beyotime, China), and the band intensities were quantified using Quantity One software (Bio-rad, USA).

### 2.8. Statistical Analysis

All statistical analyses were performed using the statistical software SPSS 13.0. Data involving more than two groups were assessed by one-way ANOVA*  * (with Games Howell test for *post hoc *analysis). Spearman correlation was used to evaluate the relationship of PEDF with insulin sensitivity. *P* values < 0.05 (2-tailed) were considered statistically significant.

## 3. Results

### 3.1. Metformin Alleviated IR in the High-Fat Diet Induced Obese Rats

**Figure 1:**
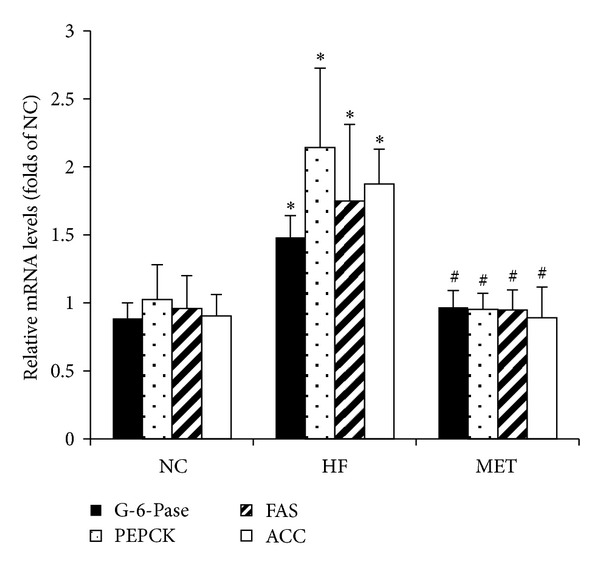
The mRNA expression of the key enzymes regulating gluconeogenesis and fatty acid synthesis in the liver. SD rats were fed a normal chow diet (NC group, *n* = 6) or a high fat diet (*n* = 12) for 15 weeks; then the high-fat-diet rats were gavaged saline (HF group, *n* = 6) or metformin 400 mg/kg/d (MET group, *n* = 6) for 4 weeks. *P < 0.05, compared with NC group. ^#^ *P* < 0.05, compared with HF group.

Compared with the NC group, rats in the HF group showed higher levels of body weight, fasting plasma glucose, fasting serum insulin, LDL-c, and a lower level of glucose infusion rate (Table 2). In addition, the mRNA expression levels of phosphoenolpyruvate carboxykinase (PEPCK) and glucose 6-phosphatase (G-6-Pase), in the liver were also significantly upregulated in the high-fat diet induced obese rats (Figure 1).

With metformin treatment for 4 weeks, the IR of the high-fat diet induced obese rats was obviously improved, manifested as enhanced glucose infusion rate, reduced serum TG and serum insulin levels, and down-regulated mRNA expression of PEPCK, G-6-Pase (Table 2, Figure 1).

### 3.2. Metformin Reduced Serum PEDF Levels in the Obese Rats and Inhibited PEDF Expression in the Adipose Tissue and Liver

**Figure 2:**
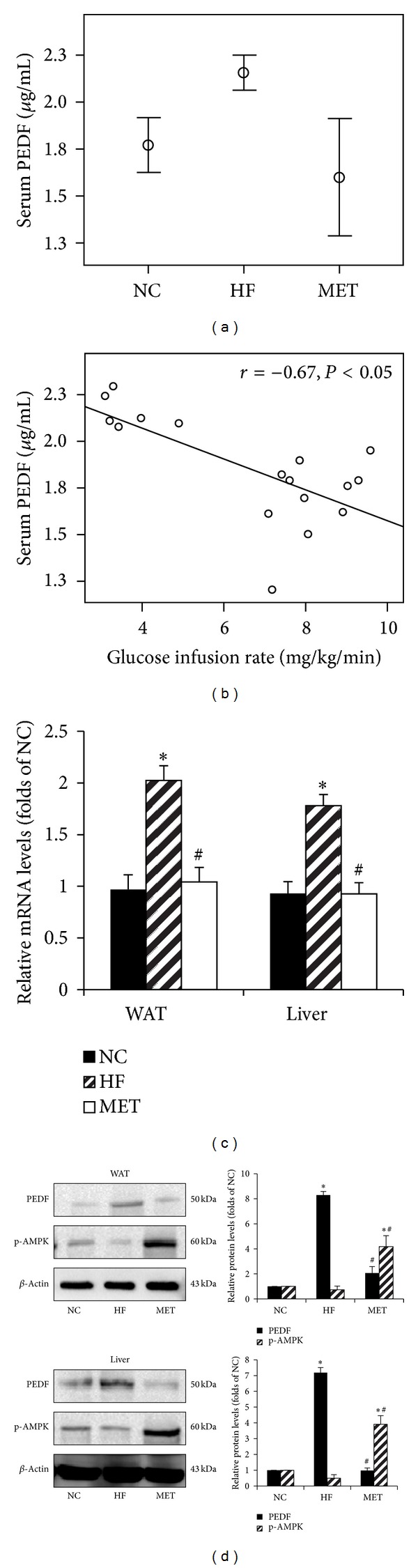
Effects of metformin on the serum PEDF levels and PEDF expression in the adipose tissue and liver of the obese rats. SD rats were fed a normal chow diet (NC group, *n* = 6) or a high fat diet (*n* = 12) for 15 weeks; then the high-fat-diet rats were gavaged saline (HF group, *n* = 6) or metformin 400 mg/kg/d (MET group, *n* = 6) for 4 weeks. At the end of the 19th week, hyperinsulinemic-euglycemic clamp were performed, and the serum, liver, and adipose tissues were collected. (a) Error bar charts showed the serum PEDF concentrations in different groups; (b) Scatter plots showed a negative correlation between serum PEDF levels and insulin sensitivity; (c) RT-PCR assay results of PEDF mRNA levels in WAT and liver; (d) Western blot analysis of PEDF protein expression and phosphorylation of AMPK. The histogram represents mean ± SD of the densitomeric scans for protein bands from three experiments, normalized by comparison with *β*-actin and expressed as a percentage of control. *P < 0.05, compared with NC group. ^#^ *P* < 0.05, compared with HF group.

The high-fat diet induced obese rats showed higher levels of serum PEDF as compared with the NC group (2.16 ± 0.09 versus 1.77 ± 0.14 *μ*g/mL, *P* < 0.05, Figure 2(a)), and a negative association was found between the serum PEDF concentration and glucose infusion rate (*r* = − 0.67, *P* < 0.05, Figure 2(b)). 

The mRNA and protein expression of PEDF in the epididymal adipose tissue and liver were significantly up-regulated in the HF group as compared to the NC group. However, the metformin group exhibited decreased serum PEDF levels (2.16 ± 0.09 versus 1.69  ±  0.24 *μ*g/mL, *P* < 0.05, Figure 2(a)) and down-regulated PEDF expression when compared with the HF group (Figures 2(c) and 2(d)).

### 3.3. Metformin also Inhibited the Expression and Secretion of PEDF in the IR Models of Adipocytes and Hepatocytes

**Figure 3:**
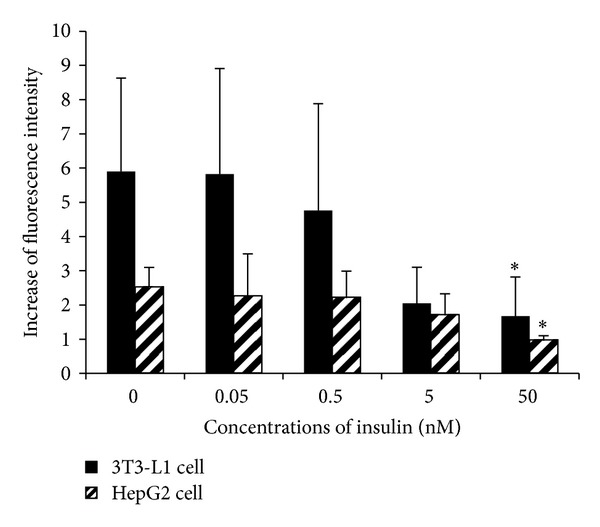
IR models of 3T3-L1 cell and HepG2 cell were established by hyperinsulinemic method (treated with 0, 0.05, 0.5, 5 and 50 nM insulin for 24 h), then stimulated by 100 nM insulin for 15 min and the fluorescence of 2-NBD labeled glucose was examined. *P < 0.05, compared with normal control group (without insulin).

To be in accordance with the IR condition in vivo, IR models of adipocytes and hepatocytes were established by the hyperinsulinemic method [16–18]. With a low or moderate concentration of insulin (0.05 nM–5 nM) treatment for 24 h, the glucose uptake in the adipocytes and hepatocytes showed no significant changes; however, when the insulin concentration was as high as 50 nM, an obvious decreased glucose uptake was observed, indicating an IR status in the cell models (Figure 3).

**Figure 4:**
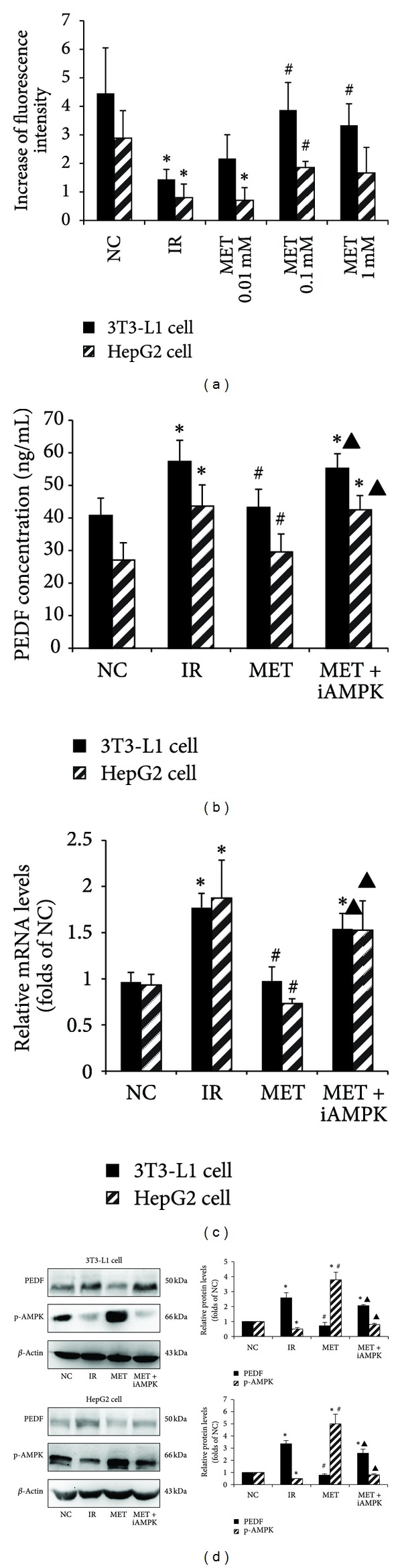
The effects of metformin on the secretion and expression of PEDF during IR improvement in vitro. (a) IR models of 3T3-L1 cell and HepG2 cell were treated with metformin (0.01, 0.1, and 1 mM) for 24 h, and 2-NBDG uptake was examined. Finally, 0.1 mM metformin was chosen for later experiments. (b) Supernatants of the cells were collected, and PEDF concentrations were determined by ELISA. (c) RT-PCR assay results of PEDF mRNA levels. (d) Western blot analysis of PEDF protein expression and phosphorylation of AMPK. The histogram represents mean ± SD of the densitometric scans for protein bands from three experiments, normalized by comparison with *β*-actin and expressed as a percentage of control. NC: normal control; IR: insulin resistance; MET: metformin; iA: inhibitor of AMPK. *P < 0.05, compared with NC group. ^#^ *P* < 0.05, compared with IR group. ^▲^ *P* < 0.05, compared with MET group.

Similar to the results in vivo, increased PEDF expression and secretion were found in the IR adipocytes and hepatocytes. While with 0.1 mM metformin treatment, the PEDF expression was down-regulated and the PEDF secretion was lowered, accompanied with increased glucose uptake in the IR adipocytes and hepatocytes (Figure 4).

### 3.4. AMPK Mediated the Effects of Metformin on PEDF

Since metformin exerts its metabolic action mainly through AMPK [3, 4], the phosphorylation at threonine 172 of the *α* subunit of AMPK was examined, and the results showed metformin treatment stimulated AMPK phosphorylation in adipose tissue and liver of the obese rats, which was accompanied with the profound reduction in PEDF protein content (Figure 2).

In vitro, when the IR adipocytes and hepatocytes were treated with metformin combined with an AMPK inhibitor compound C, the inhibitive effects of metformin on PEDF expression or secretion were abrogated (Figure 4). 

## 4. Discussion

First identified in retinal pigment epithelium cells, PEDF is expressed in various tissues throughout the body such as the eye, adipose tissue, liver, and muscle [7–9, 13]. It is reported as a multifunctional, pleiotropic protein with antiangiogenic, antioxidant, neurotrophic, and both pro- and anti-inflammatory properties [12, 13]. In the previous work, we found the serum PEDF level is elevated in women with polycystic ovary syndrome and is associated with IR, indicating PEDF may play a role in the pathogenesis of IR in patients with polycystic ovary syndrome [11].

Metformin, a widely prescribed agent to improve IR, has been reported to have an impact on the production of adipokines (e.g, adiponectin, leptin), which might be associated with the IR-improving effect of the agent [5, 6]. While PEDF has been reported to play an important role in the development of IR [7–9], it is unknown whether metformin could directly affect PEDF production and release. For the first time, our study reports that metformin inhibits the expression and secretion of PEDF in adipocyte and hepatocyte via stimulating AMPK phosphorylation, and this effect is associated with the IR-improving action of metformin.

To get insights into the effect of metformin on PEDF when metformin exerts its IR-improving action, models of IR were established. High-fat diet induced obese rats were used for the in vivo experiment and validated as being insulin resistant by the hyperinsulinemic-euglycemic clamp. In vitro, the IR models of 3T3-L1 and HepG2 cells were established by the hyperinsulinemic method and validated through glucose uptake test. It is generally accepted that IR is the most common cause of hyperinsulinemia [23]; however, recent studies have revealed that IR might also be a result of prolonged exposure of high concentrations of insulin [24–26]. Liu et al. [27] found that exposure of cultured hepatocytes to 10–100 nM insulin for 24 h induces significant IR, which might be associated with disturbed insulin signal transduction and oxidative stress. Another study reported that exposure to 1 *μ*M insulin for 6 h, 3T3-L1 cell showed down-regulated mRNA expression of glucose transporter 4 and lost their capacity for acute stimulation of glucose uptake by insulin [28]. Similar to these studies, our data revealed that, incubated with 50 nM insulin for 24 h, the adipocytes and hepatocytes were markedly insensitive to insulin as shown by the decreased glucose uptake when stimulated by 100 nM insulin. It is noteworthy that although the high concentration of 50 nM insulin induced IR in vitro, this might not be duplicated in vivo since the serum insulin level as high as 50 nM has rarely been reported in human.

Both in vivo and in vitro, increased PEDF secretion as well as expression were found in tissues and cells with IR. Furthermore, we found the serum PEDF levels were negatively correlated with the insulin sensitivity. In the study of Crowe et al. [7], an elevated serum PEDF and expression in adipose tissue were also observed in high-fat diet induced mice, which are in line with our in vivo findings. With metformin intervention for 4 weeks, high-fat diet induced obese rats exhibited obvious improvement of IR, manifested as enhanced glucose infusion rate, reduced serum TG and fasting serum insulin levels, and down-regulated mRNA expression of key enzymes which promote gluconeogenesis and fatty acid synthesis. In addition, serum PEDF levels as well as expression of PEDF in the adipose tissue and liver decreased significantly during IR improvement caused by metformin. In accordance with the in vivo findings, insulin resistant adipocytes and hepatocytes also showed decreased secretion and expression of PEDF with metformin treatment, accompanied with enhanced glucose uptake. 

To further investigate the role played by the target molecule of metformin, the phosphorylation at threonine 172 of the *α* subunit of AMPK was measured in the rats, and we found the impact of metformin on PEDF expression in adipose tissue and liver was associated with stimulation of AMPK phosphorylation. When combined with compound C, the suppressive effect of metformin on PEDF was abrogated together with the inhibited AMPK. Those results suggest that metformin exerts the effect on PEDF via stimulation of AMPK phosphorylation. Additionally, our study indicates AMPK might be a potential regulator of PEDF production, on transcriptional or posttranscriptional level, although the detailed mechanism needs further study.

The study conducted by Akin et al. [29] on 36 Turkish type 2 diabetic patients showed that, after metformin treatment for 6 months, the serum PEDF levels increased. The different subjects in Akin's study (type 2 diabetic patients) and ours (high-fat diet induced obese rats) might partially explain the discrepancy in the results of the two studies. Besides, metformin exerts complex effect in vivo, whether the increase of serum PEDF in type 2 diabetic patients is a direct effect of metformin is still unclear. Although different from metformin in mechanism, troglitazone was an insulin sensitizer which was once used to improve IR. Similar to our results, Famulla et al. [8] reported that troglitazone inhibits PEDF secretion in human primary adipocytes.

In summary, our study reveals that metformin inhibits expression and secretion of PEDF in adipocytes and hepatocytes via the stimulation of AMPK phosphorylation. In addition, our study indicates AMPK might be a potential regulator of PEDF. Although we found the effect of metformin on PEDF is closely associated with the IR-improving action of the agent, whether the action is mainly mediated through an inhibition in PEDF still needs further study.

## Figures and Tables

**Table 1:** Specific primers used for real-time PCR.

Gene	Forward and reverse primers	Amplified fragment (bp)
Actin(rat)	5′ GCTGTGCTATGTTGCCCTAGAC 3′	122
	5′ GGAACCGCTCATTGCCGATAG 3′	
Actin(mouse)	5′ GCTGTGCTATGTTGCTCTAG 3′	117
	5′ CGCTCGTTGCCAATAGTG 3′	
Actin(human)	5′ ATCGTGCGTGACATTAAG 3′	135
	5′ ATTGCCAATGGTGATGAC 3′	
PEDF(rat)	5′ CGTAGTGGAGGAGGATGAC 3′	137
	5′ TGAGAGGAGACAGCAGAATG 3′	
PEDF(mouse)	5′ CCTCAGCATCCTTCTCCTTGG 3′	117
	5′ ACTCTCACGGTCCTGTCCTC 3′	
PEDF(human)	5′ TTGAGTGGAACGAGGATG 3′	149
	5′ CTTGCCAATGAAGAGAAGG 3′	
FAS(rat)	5′ CGGCGGCAGCAGGAACAG 3′	86
	5′ GCACTCTCAGACAGGCACTCAG 3′	
ACC(rat)	5′ TTGGTGCTTATATTGTGGATGG 3′	87
	5′ ATGTGCCGAGGATTGATGG 3′	
G-6-Pase(rat)	5′ TGGTGGCTGGAGTCTTGTC 3′	81
	5′ TCTGGAGGCTGGCATTGTAG 3′	
PEPCK(rat)	5′ CCCGAAGGCAAGAAGAAATACC 3′	131
	5′ TTCATCCAGGCAATGTCATCAC 3′	

**Table 2:** Characteristics of the animal models.

	NC group (*n* = 6)	HF group (*n* = 6)	MET group (*n* = 6)
Weight at baseline (g)	218.75 ± 16.27	219.60 ± 13.78	219.60 ± 13.78
Weight at the end (g)	458.50 ± 51.50	573.30 ± 44.57*	524.2 ± 17.2*
FPG (mmol/L)	5.40 ± 0.27	6.02 ± 0.29*	5.65 ± 0.42
Fins (mIU/L)	10.98 ± 7.20	33.07 ± 12.67*	12.35 ± 7.90^#^
TC (mmol/L)	1.26 ± 0.19	1.48 ± 0.15	1.47 ± 0.22
TG (mmol/L)	0.57 ± 0.16	0.67 ± 0.24	0.31 ± 0.07^∗#^
LDL-c (mmol/L)	0.35 ± 0.10	0.48 ± 0.11*	0.52 ± 0.08*
HDL-c (mmol/L)	1.11 ± 0.20	0.97 ± 0.17	1.05 ± 0.12
GIR (*μ*mol·kg^−1^·min^−1^)	8.63 ± 0.96	3.65 ± 0.68*	7.99 ± 0.92^#^
PEDF (*μ*g/mL)	1.77 ± 0.14	2.16 ± 0.09*	1.69 ± 0.24^#^

Data are presented as mean ± SD and analyzed by one-way ANOVA with the Games Howell test for *post hoc* analysis. FPG: fasting plasma glucose; Fins: fasting serum insulin; TC: total cholesterol; TG: triglycerides; LDL-c: low-density lipoprotein cholesterol; HDL-c: high-density lipoprotein cholesterol; PEDF: pigment epithelium-derived factor; GIR: glucose infusion rate; NC: normal control; HF: high fat; MET: metformin. *P < 0.05, compared with NC group; ^#^ *P* < 0.05, compared with HF group.
